# Preclinical Evaluation of the Immunogenicity and Safety of an Inactivated Enterovirus 71 Candidate Vaccine

**DOI:** 10.1371/journal.pntd.0002538

**Published:** 2013-11-07

**Authors:** Shi-Hsia Hwa, Yock Ann Lee, Joseph N. Brewoo, Charalambos D. Partidos, Jorge E. Osorio, Joseph D. Santangelo

**Affiliations:** 1 Inviragen (Singapore) Pte. Ltd., Singapore, Singapore; 2 Inviragen Inc., Madison, Wisconsin, United States of America; 3 Department of Pathobiological Sciences, School of Veterinary Medicine, University of Wisconsin, Madison, Madison, Wisconsin, United States of America; The George Washington University Medical Center, United States of America

## Abstract

Human enterovirus 71 (EV71) is a significant cause of morbidity and mortality from Hand, Foot and Mouth Disease (HFMD) and neurological complications, particularly in young children in the Asia-Pacific region. There are no vaccines or antiviral therapies currently available for prevention or treatment of HFMD caused by EV71. Therefore, the development of therapeutic and preventive strategies against HFMD is of growing importance. We report the immunogenic and safety profile of inactivated, purified EV71 preparations formulated with aluminum hydroxide adjuvant in preclinical studies in mice and rabbits. In mice, the candidate vaccine formulations elicited high neutralizing antibody responses. A toxicology study of the vaccine formulations planned for human use performed in rabbits showed no vaccine-related pathological changes and all animals remained healthy. Based on these preclinical studies, Phase 1 clinical testing of the EV71 inactivated vaccine was initiated.

## Author Summary

Enterovirus 71 (EV71) is one of the major viruses causing Hand, Foot, and Mouth Disease, a highly contagious illness which primarily affects young children in the Asia-Pacific region and can sometimes be fatal. No vaccines or antivirals for Hand, Foot, and Mouth Disease are available at this time. We developed an experimental vaccine using inactivated, purified EV71 with an adjuvant to amplify the immune response. When this vaccine was tested in mice and rabbits, they produced large amounts of antibodies that could neutralize the virus. We were reasonably certain that the vaccine would be safe because rabbits given repeated high doses did not develop pathological lesions or clinical symptoms. Based on these results, we proceeded to test the safety of the vaccine in human adults.

## Introduction

Hand, Foot, and Mouth Disease (HFMD) is caused by several members of the human enterovirus A (HEV-A) group. It is generally a self-limiting infection affecting mostly children and is characterized by ulcers and vesicles on the hands, feet and oral cavity. However, a more severe form of disease may occur with neurological symptoms such as meningitis, encephalitis, polio-like paralysis, and brain stem encephalitis leading to pulmonary edema and death [1]–[3]. Since the mid-1990s, HFMD infections caused by human enterovirus 71 (EV71) have resulted in significant morbidity and mortality, particularly in the Asia-Pacific region [4], [5]. China, Viet Nam, and Singapore reported increased activity in January–May 2012 compared to the same period in 2011, including 22 deaths to date in Viet Nam [6]. In addition, HFMD outbreaks disrupt education and economic activities due to school and childcare center closures in efforts to control disease transmission [7].

Human enterovirus A belongs to the *Picornaviridae* family of non-enveloped, positive-sense RNA viruses, which also includes polioviruses and rhinoviruses. Members of the HEV-A group which cause HFMD include EV71 and Coxsackievirus A16 (CAV16) [8]. HFMD outbreaks due to EV71 infection have the greatest propensity to cause severe neurological disease. Experimental infection of cynomolgus macaques showed that strains isolated across several decades were all neurotropic, as well as showing a broader tissue tropism than polioviruses [9].

Enterovirus 71 has four capsid proteins (VP1–VP4) and seven nonstructural proteins. In addition to protecting the viral RNA, the capsid proteins recognize receptors on the surface of host cells and contribute to the antigenic profile of the virus [8]. Known human cell surface receptors for EV71 are the scavenger receptor B2 (SCARB2), and the P-selectin glycoprotein ligand 1 (PSGL-1) [10], [11].

Although the classical method of typing enteroviruses by serum neutralization defines EV71 as a single serotype [12], current molecular typing methods reveal that several genogroups have been circulating in the Asia-Pacific region at least since the 1990s [13]. EV71 isolates were previously classified into genogroups A, B, and C and sub-genogroups based on VP1 nucleotide sequences alone [14]; nucleotide sequence identity of the VP1 gene is >92% within genogroups, whereas nucleotide sequence identity between the genogroups is 78–83% [4]. However, whole-genome sequencing resulted in the reclassification of subgenogroup B5 under B4 and addition of genogroup D; the authors suggested that the 3D polymerase sequence together with VP1 better represented whole genomes [15]. Recombination between genogroups and with other Human enterovirus A serotypes also occurs [16].

At present there is no specific antiviral therapy or vaccine available against EV71. Intravenous immunoglobulin has been used in severe HFMD cases, with some therapeutic benefit suggested by the outcomes but as yet unproven by clinical trials [3], [17]. Preventive and control measures during EV71 outbreaks are limited to surveillance, closure of educational and childcare facilities, and isolation of patients. Candidate vaccines under development include formaldehyde-inactivated whole-virus vaccine [18], [19], VP1 subunit vaccines [20], [21], a peptide-based synthetic vaccine [22], a recombinant bacterial-vectored VP1-based vaccine [23], a plasmid DNA vaccine expressing VP1 [24], virus-like particles of EV71 [25], and a live –attenuated vaccine [26]. Common findings include the necessity for an adjuvant [27], and the use of whole virus particles as opposed to recombinant proteins or short peptides alone [28]. Human clinical trials of inactivated virus vaccines producing high neutralizing antibody titers have been conducted in China [29], [30], Taiwan [31], and Singapore (Inviragen, manuscript in progress).

In the following studies, we describe the development of an inactivated EV71 candidate vaccine in animal models and evaluation of its immunogenicity and safety in preparation for human clinical trials.

## Materials and Methods

### Ethics statement

Animal studies were performed at the following institutions and approved by the respective animal care and use committees: Alhydrogel adjuvant study at University of Wisconsin-Madison (approved by UW-Madison Institutional Animal Care and Use Committee, under USDA Animal Welfare Act); determination of optimum immunogenic dose at Shanghai Genomics (under Chinese regulatory guidelines); rabbit GLP (Good Laboratory Practice) toxicology study at Jai Research Foundation (approved by JRF Institutional Animal Ethics Committee, under “Guidelines for Laboratory Animals Facility”, CPCSEA, India).

### Viruses and cell culture

The genogroup B EV71 strain, MS/7423/87 (GenBank accession number U22522.1) was selected to prepare the candidate vaccine on the basis of amino acid sequence similarity to highly immunogenic strains as well as high yield in Vero cell culture. The candidate vaccine strain was grown in Vero cells (WHO Reference Cell Bank 10-87) in 10-tier cell factories as described below, using Dulbecco's Modified Eagle's Medium (DMEM; Sigma-Aldrich, United States) without serum. Small scale preparations for neutralization assays were grown in Vero clone E6 cells (ATCC CRL-1586) in tissue culture flasks (BD Biosciences, United States) using Minimum Essential Medium (MEM; Sigma-Aldrich, United States) with 2% fetal bovine serum (FBS; Biological Industries, Israel).

### Inactivated EV71 virus

Confluent monolayers of Vero cells in 10-tier cell factories (Nalge Nunc International, United States) were infected with the EV71 vaccine strain at a multiplicity of infection (MOI) of 0.1 to 0.01. When cytopathic effect (CPE) was complete, the medium was harvested, clarified by filtration, followed by treatment with benzonase (Merck Chemicals, Germany) to remove host cell nucleic acid at 20 U/mL, 37°C for 24 hours. The clarified harvest was treated with freshly prepared “binary” ethyleneimine (BEI) at 1.5% v/v, 37°C for 6 hours to inactivate EV71 infectivity [32], followed by the addition of 150 mM sodium thiosulfate to hydrolyze unreacted ethylenimine. Inactivation of viral infectivity was confirmed at this stage by blind-passaging the material twice on Vero cells. Tissue culture medium components and low molecular weight proteins were partially removed by concentration and diafiltration against phosphate-buffered saline (Invitrogen, United States) containing 0.002% Tween 80 (Merck Chemicals, Germany) (PBST). The inactivated virus preparation was further purified by chromatography using ion-exchange and size-exclusion columns (details of the chromatography process are proprietary), concentrated using a 30 kDa molecular weight cut-off membrane, and finally sterilized by filtration through a 0.2 µm membrane. Purified inactivated EV71 antigen was stored at −80°C in suspension in PBST.

### Characterization of purified inactivated EV71 antigen

The presence and estimated purity of EV71 antigen in samples were evaluated by SDS-PAGE and Western blotting. Briefly, samples were heated to 95°C in lithium dodecyl sulfate buffer (Invitrogen) with beta-mercaptoethanol (Sigma-Aldrich) and fractionated on duplicate 4–12% PAGE gels (Invitrogen) in MES-SDS buffer. For purity estimation, one gel was stained with colloidal blue (Invitrogen) followed by image intensity calculations using Quantity One software (Bio-Rad). For detection of EV71 antigens, proteins were transferred to a PVDF membrane using the iBlot semi-dry blotting system (Invitrogen) and stained for the presence of VP2 and VP0 using the monoclonal antibody 422-8D-4C-4D (Merck Millipore, catalogue number MAB979), followed by an HRP-conjugated anti-mouse IgG secondary antibody (DAKO) and DAB substrate (Sigma-Aldrich) to visualise bands.

The physical form of the EV71 antigen present was examined by transmission electron microscopy using a phosphotungstic acid negative stain on carbon formvar grids (Electron Microscopy Services).

### Immunization procedures

In the two following mouse studies, male BALB/c mice between 4–6 weeks old were used. Volumes of 100 µL per dose were administered by intramuscular (IM) injection in the hind leg as two injections of 50 µL in the same leg, due the limitations on the volume of a single injection in mice.

Immunogenicity of EV71 antigen formulated with or without aluminum hydroxide: Groups of mice (n = 8) were injected on Days 0 and 28 with the following doses of purified inactivated EV71 antigen (0.12 µg, 0.6 µg, or 3.0 µg) in PBST only or with aluminum hydroxide (Alhydrogel “85”, Brenntag Biosector, Denmark) at 0.5 mg (aluminium content) per dose. Two groups of control animals (n = 8) were injected with PBST or Alhydrogel (0.5 mg per dose) using the same immunization protocol as above. Blood samples were collected on day 0, 28, 42, 56, 91, and 120; sera were stored at −20°C until testing for neutralizing activity.

Determination of the optimum immunogenic dose: Groups of mice (n = 10) were immunized on days 0 and 28 with the following doses of purified inactivated EV71: 0.12 µg, 0.6 µg, 3 µg, 9 µg, and 15 µg, formulated with Alhydrogel (0.5 mg in a volume of 100 µL per dose). A control group received Alhydrogel alone (0.5 mg per dose in a volume of 100 µL per dose). Blood samples were collected on day 0, 28, and 56; sera were stored at −20°C until testing for neutralizing activity.

The toxicology study was conducted according to Good Laboratory Practice (GLP) at the Jai Research Foundation, India. New Zealand White rabbits were used as the test species with females to males at a 1∶1 ratio in each group. Low and high dose vaccines were formulated as planned for a Phase I human clinical trial, containing 0.6 µg and 3.0 µg EV71 antigen respectively, with 0.5 mg Alhydrogel in a 0.5 mL volume. Twenty rabbits received each vaccine formulation. Control groups received normal saline (PBS, n = 12) or 0.5 mg Alhydrogel alone (vaccine placebo, n = 16). Test articles were administered by IM injection in the left thigh. All animals received a booster injection of the same dose and route on day 28; the animals in the high dose group received a second booster on day 42.

Animals were observed for morbidity and mortality at least twice daily during the treatment and observation periods, in addition to weekly clinical examinations. Parameters monitored were: Injection site reactogenicity (Draize numerical scoring system), rectal temperature, body mass, food consumption (grams/rabbit/week), ophthalmological examination, and respiratory rate. Half the animals of each sex in each group were sacrificed 2 days after the last immunization, either on Day 30 (low dose, saline placebo, and Alhydrogel placebo groups) or Day 44 (high dose group); the remaining animals were sacrificed on Day 56. Blood samples were collected on Days 0, 3, 28, 42 (high dose group only), and 56 and used for clinical chemistry, hematology, and EV71 neutralizing antibody assays. Gross pathology examinations were performed on all sacrificed animals. A complete necropsy and histopathology examinations were performed on organs from the animals sacrificed on Day 30 (saline placebo, Alhydrogel placebo, and vaccine low dose groups) or Day 44 (high dose group).

### Determination of neutralizing antibody responses

Vero cells were seeded into 96-well microtiter plates at 10^4^ cells per well in 100 µL growth medium (MEM+10% FBS). Individual serum samples were heat-inactivated at 56°C for 30 min. Two-fold serial dilutions of serum samples in assay medium (MEM+2% FBS) were mixed with equal volumes of an EV71 (vaccine strain) suspension at 2000 units of 50% tissue culture infectious dose (TCID_50_) per mL and incubated at 37°C for 1.5 hours. 100 µL of each serum-virus mixture was added to three wells (final virus titre 100 TCID_50_ per well). Each well was scored for CPE at 5 days post-infection. The end-point neutralizing titer was defined as the highest serum dilution in which at least two of the three replicates were negative for CPE. Seroconversion was defined as a reciprocal neutralizing titer ≥128 [33].

### Statistics

Data from animal studies were compiled in Microsoft Excel and analyzed using Excel or Prism 5 (GraphPad, Inc). Student's t-test was used on log-transformed reciprocal neutralizing titers to compare immunogenicity between groups. *P* values ≤0.05 were considered significant.

## Results

### Characterization of purified inactivated EV71 material

**Figure 1:**
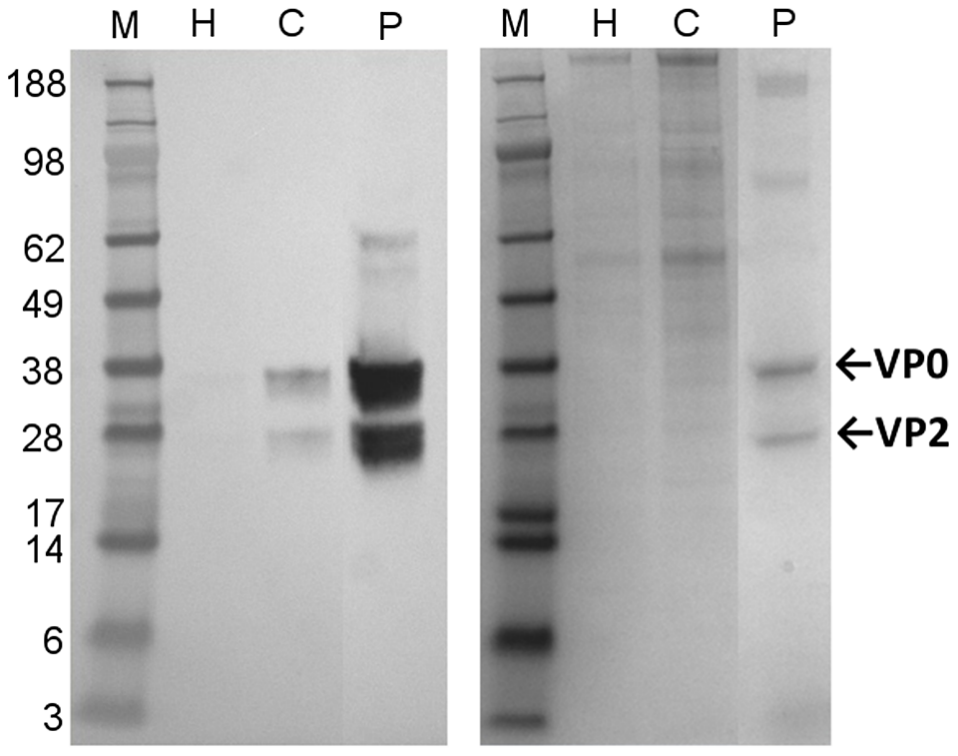
SDS-PAGE and Western blotting of purified inactivated EV71 antigen. Left: Western blot of inactivated harvest (H), concentrated inactivated harvest (C), and concentrated purified inactivated material (P). VP0 is found at 38 kDa and VP2 at 28 kDa. Right: Colloidal blue stain of the same samples. Molecular weight marker (M) is SeeBlue Plus2 (Invitrogen).

Western blotting with a monoclonal antibody specific for EV71 VP2 and VP0 proteins confirmed the presence of EV71 antigen in purified inactivated material (samples from a typical batch shown in Figure 1, left). The estimated purity of EV71, based on image intensity measurements of colloidal blue-stained gels (same samples in Figure 1, right), was >80% in all batches using this process, typically 85–90% (calculations not shown). Typical yields from the process outlined above were ≈300 µg of protein per liter of harvest.

**Figure 2:**
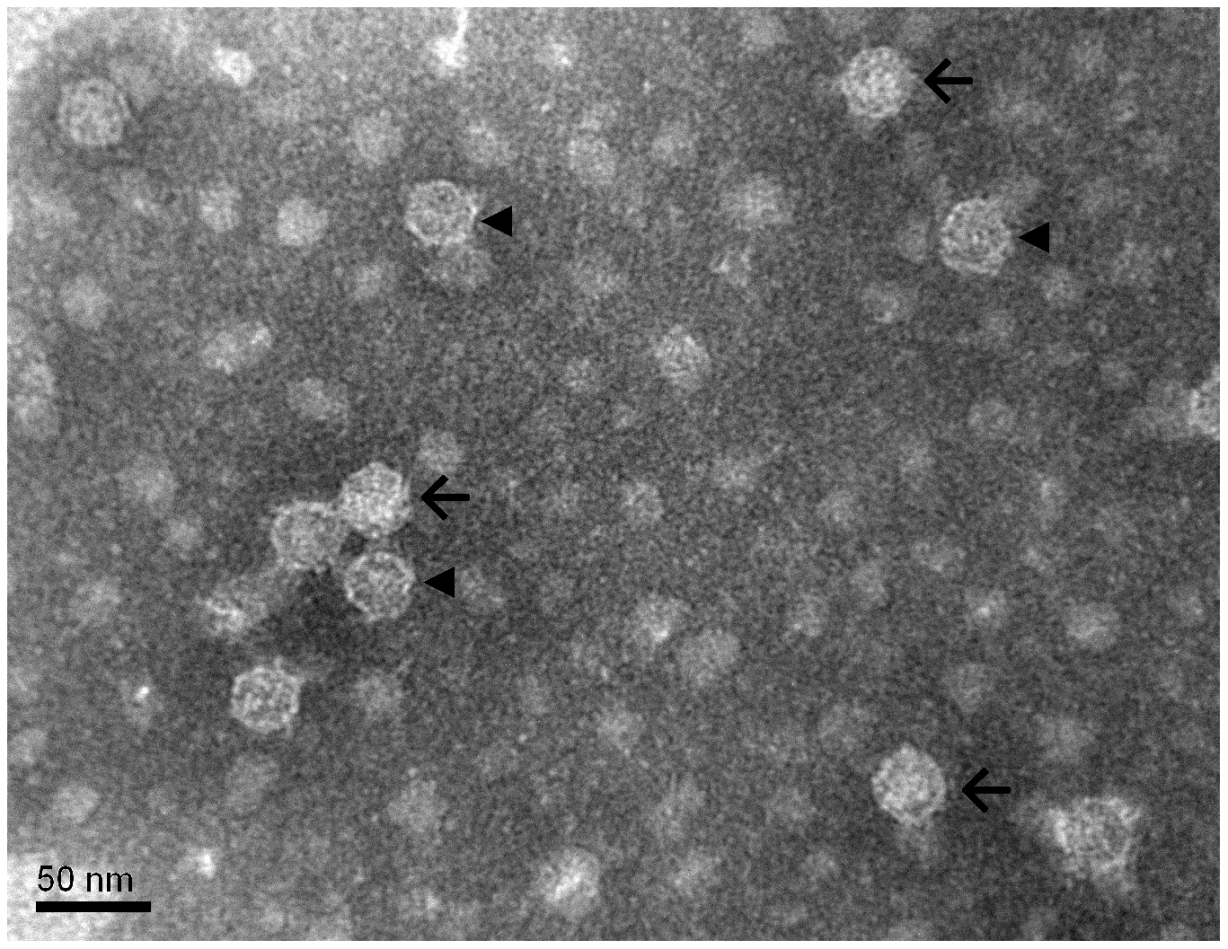
Transmission electron micrograph of purified inactivated EV71. Purified inactivated EV71 material is shown on a carbon formvar grid with phosphotungstic acid negative staining. Examples of full particles are indicated with arrows; examples of empty particles are indicated with arrowheads. The scale bar is 50 nm.

Electron microscopy revealed a mixture of filled and empty icosahedral capsids (Figure 2), consistent with the observation of VP0 in Western blotting. A particle concentration estimate performed by mixing the purified inactivated EV71 with a polystyrene bead standard (micrograph not shown) was approximately 3E+10 particles/mL.

### Immunogenicity of EV71 antigen formulated with or without aluminum hydroxide

In a preliminary experiment we compared the immunogenicity of EV71 antigen inactivated by heat (56°C for 6 hours) or chemically (BEI) in mice. The BEI-inactivated EV71 virus exhibited superior immunogenicity, inducing ∼6.4-fold higher neutralizing titres at 28 days post-immunization; the difference decreased to ∼1.5-fold higher at 56 days post-immunization (data not shown).

**Figure 3:**
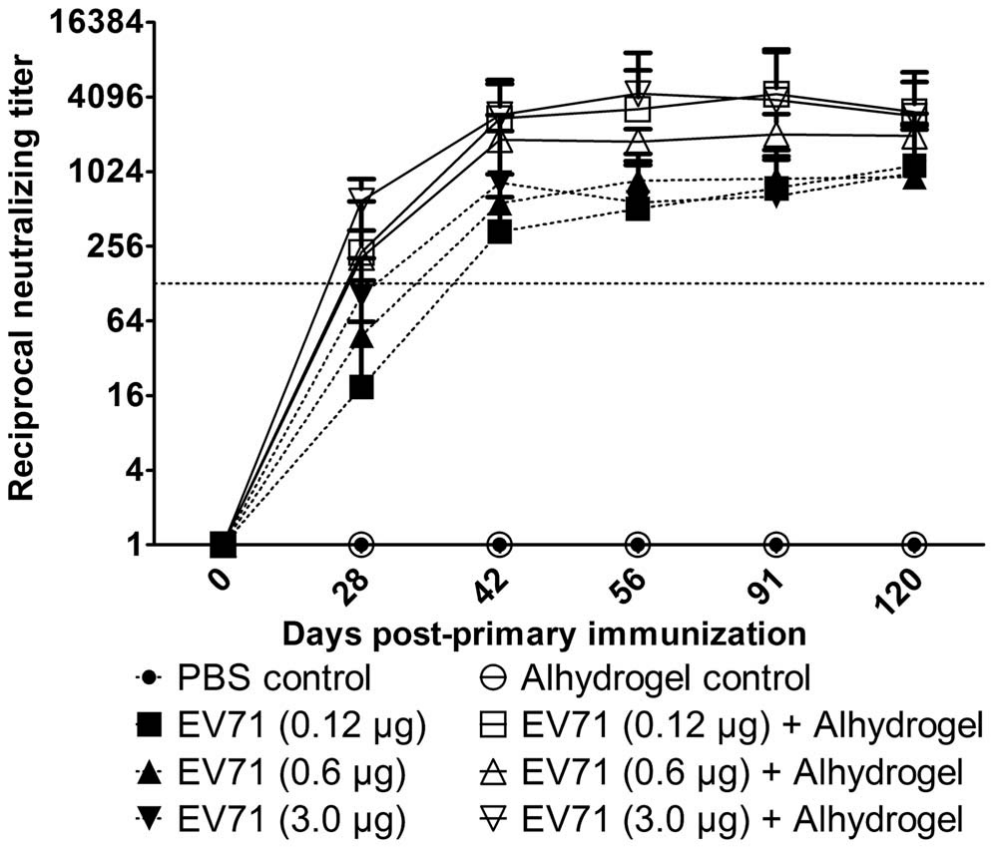
Kinetics of serum neutralizing antibody responses to EV71 with or without adjuvant. Groups of BALB/c mice (n = 8) were immunized with purified inactivated EV71 with or without Alhydrogel, PBST-only control, or Alhydrogel control. Symbols represent mean of reciprocal neutralizing antibody titers from groups of 8 animals ± standard deviation. Y-axis is plotted on a logarithmic scale. Dotted line shows titer of 128 (7 log_2_).

Subsequently, we tested the immunogenicity of BEI-inactivated, purified EV71 preparations in mice at different concentrations with or without adjuvant. The presence of aluminum hydroxide produced higher seroconversion rates at 28 days after the first dose (Table 1). Neutralizing antibody titers in groups receiving antigen with adjuvant, compared to groups receiving antigen alone ranged from 11-to-23-fold higher at day 28 (after 1 dose), 4-to 6-fold higher at day 42 (after 2 doses) and 2.4-to 9-fold higher at day 56 (two weeks after 2nd dose) (Figure 3). The differences were significant at all dose levels (*P*<0.05). Follow-up to 91 and 120 days showed that high titers were sustained and did not significantly decrease from Day 56 (Figure 3).

**Table 1:** Seroconversion rates in BALB/c mice immunized with purified inactivated EV71.

		Day 28	Day 42	Day 56
Group	Treatment	% seroconverted (n)	% seroconverted (n)	% seroconverted (n)
1	PBST^a^ control	0	0	0
2	0.12 µg EV71	12.5 (1/8)	100 (8/8)	100 (8/8)
3	0.6 µg EV71	12.5 (1/8)	87.5 (7/8)	100 (8/8)
4	3 µg EV71	37.5 (3/8)	100 (8/8)	100 (8/8)
5	Alum control	0	0	0
6	0.12 µg EV71+Alum	37.5 (3/8)	100 (8/8)	100 (8/8)
7	0.6 µg EV71+Alum	100 (8/8)	100 (8/8)	100 (8/8)
8	3 µg EV71+Alum	100 (8/8)	100 (8/8)	100 (8/8)

a PBST = Phosphate Buffered Saline + 0.002% (v/v) Tween 80, buffer base for purified inactivated EV71.

Mice were immunized with inactivated virus with or without Alhydrogel at 28, 42 and 56 days post primary immunization. Seroconversion is defined as a neutralizing antibody titre of ≥128.

### Determination of the optimum immunogenic dose

**Figure 4:**
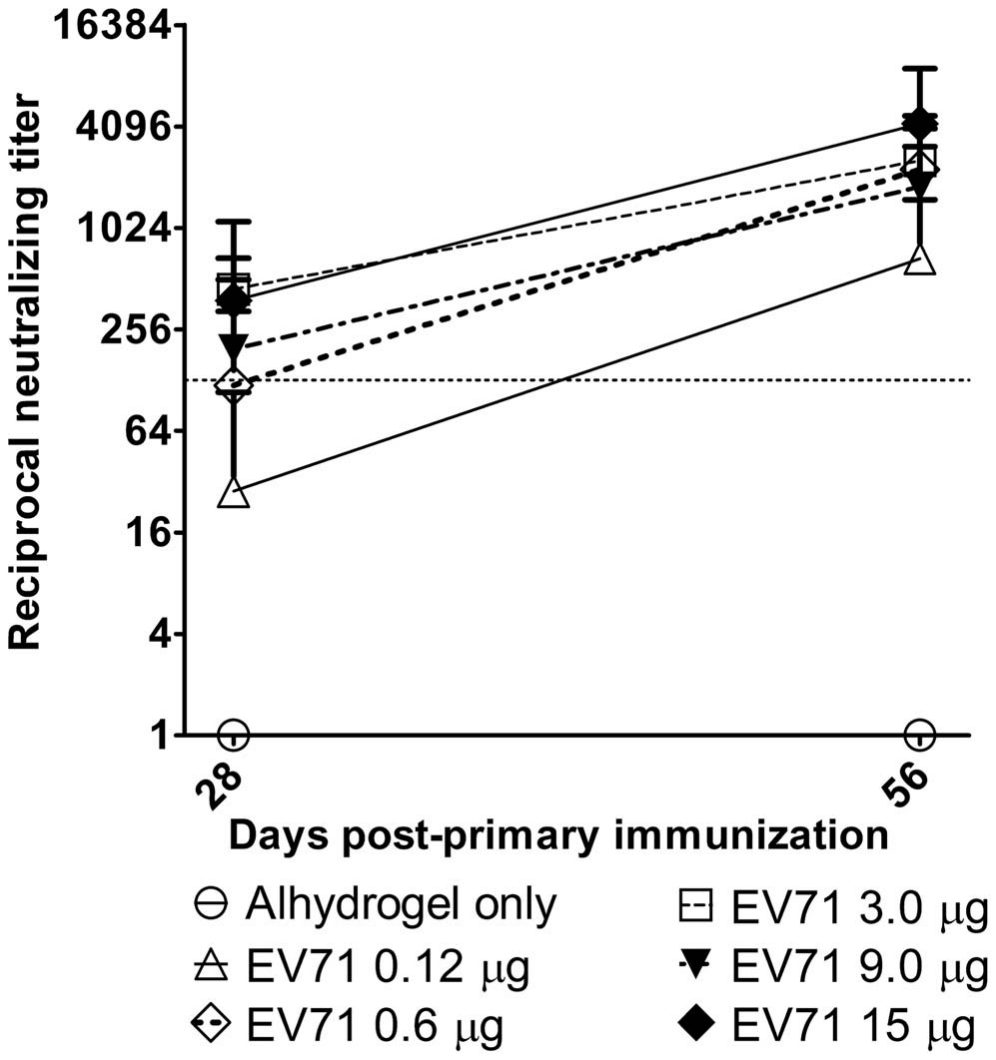
Dose titration of EV71 antigen. Groups of BALB/c mice (n = 10) were immunized with purified inactivated EV71 with Alhydrogel, or Alhydrogel control. Symbols represent mean of reciprocal neutralizing antibody titers from groups of 10 animals ± standard deviation. Y-axis is plotted on a logarithmic scale. Dotted line shows titer of 128 (7 log_2_).

Following a single dose, only the 3 µg and the 15 µg dose levels elicited a high neutralizing antibody response by day 28 (Figure 4). Seroconversion rates for all groups were incomplete, ranging from 10% to 80% depending on the dose (Table 2). However, a booster immunization induced significantly (*P*≤0.05) higher levels of neutralizing antibodies in all groups of animals (Figure 4). Twenty-eight days after the boost, only the lowest dose of 0.12 µg was significantly less immunogenic than the highest dose of 15 µg; doses from 0.6 µg and higher produced equivalent titers. In addition, 100% seroconversion was observed following the booster dose in animals receiving vaccine doses of 3 µg or higher (Table 2).

**Table 2:** Seroconversion rates following varying doses of purified inactivated EV71.

		Day 28	Day 56
Group	Dose	% seroconverted (n)	% seroconverted (n)
1	Alum control	0 (0/10)	0 (0/10)
2	0.12 µg EV71+Alum	10 (1/10)	50 (5/10)
3	0.6 µg EV71+Alum	20 (2/10)	90 (9/10)
4	3 µg EV71+Alum	70 (7/10)	100 (10/10)
5	9 µg EV71+Alum	40 (4/10)	100 (10/10)
6	15 µg EV71+Alum	80 (8/10)	100 (10/10)

BALB/c mice were immunized with purified inactivated EV71 virus with Alhydrogel, at 28 days and 56 days post primary immunization. Seroconversion is defined as a neutralizing antibody titre of ≥128.

### Safety of the EV71 candidate inactivated vaccine

A toxicological study using this animal model to test low and high dose vaccine formulations was conducted at a GLP contract research organization (CRO). Clinical and histological findings were compiled by the CRO and reported to Inviragen. No morbidity or mortality occurred in any animals, and no skin reactions (i.e. no erythema or edema) at the site of injection were found in any animals. Hematology and clinical chemistry parameters remained within the normal range for NZW rabbits. Animals in the EV71 vaccine-treated groups exhibited no differences from the control groups in group averages of body mass, body mass change during the study period, or food consumption. At day 30, males treated with Alhydrogel placebo had higher thymus weights compared to the saline placebo group. At day 56, EV71 vaccine-treated females had higher adrenal, ovary/oviduct, and uterus weights compared to the Alhydrogel placebo group. However, no lesions of pathological significance were found on the reproductive or other internal organs.

Muscular degeneration at the site of injection, with or without mononuclear cell/eosinophil infiltration, were observed in a sub-population of animals in all groups receiving formulations containing aluminum hydroxide including the Alhydrogel placebo group, but not in animals which received the saline placebo (Table 3). The lesions had healed or were markedly reduced by day 56, i.e. 28 days after the last immunization for the Alhydrogel placebo and low dose groups, and 14 days after the last immunization for the high dose group.

**Table 3:** Muscular lesions at the site of injection in rabbit toxicological study, 2 days after the last immunization.

Group	Treatment	Dose of EV71 (µg)	Muscle Degeneration (animals/group)		
			Without Infiltration	With Infiltration	Total^a^
G1	Saline Placebo	0	0/12	0/12	0/12
G2	Alhydrogel Placebo^b^	0	4/16	2/16	6/16
G3	Vaccine Low Dose	0.6	4/20	6/20	10/20
G4	Vaccine High Dose	3.0	0/20	8/20	8/20

a Values shown under total are the total number of observations of muscle degeneration with and without MNC/eosinophil infiltration.

b The Alhydrogel Placebo group received an Alhydrogel-only adjuvant formulation.

## Discussion

Prompted by the successful control of poliovirus by formalin-inactivated vaccines, we produced a purified, inactivated, aluminum hydroxide-adjuvanted EV71 candidate vaccine. The inactivation method used leaves no detectable residual inactivant and completely eliminates viral infectivity within <24 hours while preserving antigenicity (data not shown), thus is more efficient than formalin inactivation, which takes several days at 37°C [34]. BEI also better preserves the immunogenicity of the viral particles compared to heat inactivation, possibly because heating EV71 to ≥50°C triggers a conformational change in the particles [35], [36]. The purification process using ion-exchange and size-exclusion chromatography does not separate empty procapsids from RNA-containing particles, or immature virions with VP0 from mature virions with cleaved VP2/VP4, therefore the purified inactivated antigen preparations contain a mixed population of particles. However, empty particles from other EV71 candidate vaccine strains are known to be immunogenic albeit inducing somewhat lower neutralizing titres than full particles, the differences in immunogenicity being lesser at higher doses [34].

We then evaluated immunogenicity of several vaccine formulations using this antigen preparation in mice, followed by a repeated-dose toxicology study in rabbits. The route of administration, dose levels, dosing schedule, and selection of parameters to be monitored in the study were designed according to industry standard recommendations for evaluation of vaccine safety [37]; furthermore, the high dose group received an additional dose beyond the planned human dosing schedule. No clinical or hematological abnormalities, or pathological signs on internal organs were found. Injection site lesions were localised, microscopic, and transient, and consistent with aluminium salt-induced lesions reported by other investigators [38]. These lesions occurred in the Alhydrogel placebo group as well as the low and high dose EV71 vaccine groups, therefore they were considered to be due to the Alhydrogel adjuvant rather than EV71 antigen.

The purified inactivated EV71 antigen preparation was shown to elicit neutralizing antibody responses to the homologous virus in mice, which were significantly increased by the addition of aluminum hydroxide adjuvant. Analysis of the neutralizing antibody responses induced by the various antigen concentrations established i) the requirement of two doses for optimum immunogenicity and reliable seroconversion, and ii) the selection of low (0.6 µg antigen per dose) and high (3 µg) doses. Currently, the protective anti-EV71 neutralizing antibody titer has not been determined for humans. However, it is interesting to note that the neutralizing antibody titers elicited by the inactivated vaccine in animals were well over the target titer of 128 and were sustained at high levels for over 3 months after the last immunization. This titer was the minimum protective antibody dose in a neonatal mouse passive immunization-challenge model of EV71 infection [33]. Subsequent to the studies described in this paper, a Phase I human clinical trial was conducted in adult volunteers (Inviragen, manuscript in progress).

A significant challenge in the development and evaluation of candidate EV71 vaccines has been the lack of a suitable animal model that mimics EV71 pathogenesis in humans. Although nonhuman primates are susceptible to EV71 infection [9], [26], cost and animal welfare considerations are barriers to use. Some neonatal mouse models have been established [39]–[41], but most strains of mice become resistant to EV71 infection by a few days of age. The interferon receptor-deficient mouse strain AG129 has been found by other investigators to be susceptible to EV71 infection [42]. The candidate vaccine described in this paper was later tested in the AG129 model in both passive transfer and vaccination studies, demonstrating its efficacy for protecting against EV71 disease [43]. Adult mice that were immunized with 3 µg EV71 in Alhydrogel (antigen dose equivalent to the planned clinical high dose) were partially protected against morbidity and mortality following a primary vaccination, and fully protected following one booster.

We did not extensively test cross-neutralization of heterologous strains of EV71 by animal serum *in vitro*; however, preliminary data from the Phase I clinical trial of this candidate vaccine showed increases following vaccination of neutralizing titers against the genogroup B2 vaccine strain in addition to genogroup B4, C2, C4, and C5 isolates (Inviragen, manuscript in progress). While neutralizing titers against heterologous strains may vary, there is not a straightforward relationship between antigenicity and the current system of genotyping especially among genogroup B and C strains [18], [44], as some neutralizing epitopes appear to be conformational rather than linear [45]. Antisera raised against various strains have been reported to be broadly cross-neutralizing [18], [46], [47]. Molecular studies also show that isolates across all EV71 genogroups share high predicted amino acid sequence identity including at the immunodominant neutralizing epitope in VP1 [14], [28].

The development of an inactivated EV71 vaccine has three potential advantages over live attenuated vaccines: i) unlike live attenuated vaccines, the virus cannot revert to a more pathogenic phenotype; ii) immune responses can be manipulated (e.g. T_h_1 vs. T_h_2) by the inclusion of appropriate adjuvant; and iii) production will be cost-effective and flexible since the virus can be easily grown to high titers in tissue culture cells at scale. The decision to introduce an EV71 vaccine in developing countries with limited financial resources will depend on the balance of the economic value of EV71 vaccination versus EV71 risk. For instance, a recent study has estimated that in China, based on the current EV71 risk, a vaccine with 70% efficacy would be cost-effective at USD 25 per dose for mass immunization [48]. Encouraging results from a Phase 3 study show that high efficacy is in fact achievable with an inactivated vaccine [30]. Efficacy of an inactivated vaccine against HFMD could be further improved by inclusion of a coxsackievirus A16 (CVA16) strain. CVA16 is another major cause of HFMD and cross-neutralization of CVA16 by anti-EV71 antiserum is low, likely due to sequence divergence at the VP1 neutralizing epitope and different conformation of the virion [28].
